# Normal Motor Adaptation in Cervical Dystonia: A Fundamental Cerebellar Computation is Intact

**DOI:** 10.1007/s12311-014-0569-0

**Published:** 2014-05-29

**Authors:** Anna Sadnicka, Bansi Patani, Tabish A. Saifee, Panagiotis Kassavetis, Isabel Pareés, Prasad Korlipara, Kailash P. Bhatia, John C. Rothwell, Joseph M. Galea, Mark J. Edwards

**Affiliations:** 1 Sobell Department for Motor Neuroscience, UCL Institute of Neurology, 33 Queen Square, London, WC1N 3BG UK; 2 School of Psychology, University of Birmingham, Birmingham, UK

**Keywords:** Spasmodic torticollis, Cerebellum, Forcefield adaptation, Visuomotor adaptation, Motor control

## Abstract

The potential role of the cerebellum in the pathophysiology of dystonia has become a focus of recent research. However, direct evidence for a cerebellar contribution in humans with dystonia is difficult to obtain. We examined motor adaptation, a test of cerebellar function, in 20 subjects with primary cervical dystonia and an equal number of aged matched controls. Adaptation to both visuomotor (distorting visual feedback by 30**°**) and forcefield (applying a velocity-dependent force) conditions were tested. Our hypothesis was that cerebellar abnormalities observed in dystonia research would translate into deficits of cerebellar adaptation. We also examined the relationship between adaptation and dystonic head tremor as many primary tremor models implicate the cerebellothalamocortical network which is specifically tested by this motor paradigm. Rates of adaptation (learning) in cervical dystonia were identical to healthy controls in both visuomotor and forcefield tasks. Furthermore, the ability to adapt was not clearly related to clinical features of dystonic head tremor. We have shown that a key motor control function of the cerebellum is intact in the most common form of primary dystonia. These results have important implications for current anatomical models of the pathophysiology of dystonia. It is important to attempt to progress from general statements that implicate the cerebellum to a more specific evidence-based model. The role of the cerebellum in this enigmatic disease perhaps remains to be proven.

## Introduction

Primary dystonia is an enigmatic disease, which since its original description in 1911 has provoked lively debate surrounding its definition and pathophysiology [1, 2]. For many years dystonia was conceptualised as a basal ganglia disorder; however, recently, a case has been made for cerebellar involvement within a growing dystonic sensorimotor network [3, 4]. In rodent models modulating cerebellar function can cause or abolish dystonia (4–6). In humans, pathology of the cerebellum can produce secondary dystonia and there is a growing literature linking cerebellar dysfunction to primary dystonia (7). However, this area of research is still at an early phase and defining the extent and nature of cerebellar involvement in patients with primary dystonia is incomplete.

In the motor control literature, the archetypal cerebellar-dependent paradigm is adaptation. This paradigm requires subjects to adapt their performance of a task (such as reaching to hit a target) after an environmental perturbation (such as distortion of visual feedback) introduces a movement error. The sensory prediction error (how the actual sensory movement outcome differed from the predicted sensory movement outcome) is used to update subsequent motor performance, with this type of learning being strongly dependent on the cerebellum [5]. For example, the ability to adapt behaviour in response to a novel perturbation is impaired in patients with cerebellar lesions [6–8]. It is thought that the cerebellum is crucial for the formation of forward models, which predict the sensory consequences of a motor command and drive adaptation [9]. Thus, the ability to adapt has direct relevance to the clinical manifestation of dystonia and if impaired would provide a valuable model of how the cerebellum contributes to the pathophysiology of dystonia.

We have examined the ability to adapt as a marker of cerebellar function in 20 subjects with primary cervical dystonia and an equal number of aged matched controls. A purpose built robotic arm enabled detailed kinematic analysis of arm movements, and we have tested adaptation to both visuomotor and forcefield perturbations for which visual and proprioceptive afferent feedback dominate, respectively [10, 11]. Our hypothesis was that cerebellar abnormalities observed in dystonia research would translate into deficits of cerebellar adaptation. We also examined the relationship between adaptation and dystonic head tremor as many primary tremor models implicate the cerebellothalamocortical network which is specifically tested by this motor paradigm [12].

## Methods

### Subjects

Twenty patients with primary cervical dystonia were recruited from the National Hospital for Neurology and Neurosurgery, London (Table 1). Patients were tested at least 3 months after their last botulinum toxin treatment, and none were taking oral medications for dystonia. Twenty age-matched controls were also recruited. Subjects did not have any additional neurological or musculoskeletal problems of the arm or significant cognitive impairment. Written informed consent was obtained from all participants. The study had been approved by the local ethics committee.

**Table 1:** Clinical characteristics of patients. The severity subscore of the TWSTRS is out of 35. The total TWSTRS which also incorporates disability and pain subscores is out of a total of 87

Age	Symptomatic head tremor?	TWSTRS		Tremor	
		Severity subscore	Total	Frequency (Hz)	Power
56	Yes	6	38	NA	NA
76	Yes	2	12	NA	NA
68	Yes	24	44	5.8	85.68
53	No	18	27	5.6	2.11
61	No	15	23	6.6	0.08
75	Yes	2	11	3.1	17.78
63	No	2	14	4.8	0.08
39	No	13	16	6.7	2.40
40	No	19	40	4.3	0.08
40	No	20	43	4.9	0.11
61	Yes	16	44	5.5	14.37
66	No	8	16	7.1	1.07
69	Yes	17	35	3.5	1.02
71	Yes	18	61	3.7	0.39
80	Yes	9	44	3.8	2.83
57	Yes	20	24	4.1	2.14
51	Yes	24	56	7.5	2.48
67	Yes	16	21	3.5	7.20
53	Yes	11	27	4.9	5.59

### Clinical Assessment

Severity of cervical dystonia was examined using the Toronto Western Spasmodic Torticollis Rating Scale (TWSTRS). Head tremor was objectively captured by tri-axial accelerometry prior to the adaptation task with a commodity mobile communication device (HTC Desire) at a sampling frequency of 100 Hz and analysed off-line. The device was strapped to the head below the occipital protuberance. Tremor recordings were made for 30 s. Data were analysed with Spike software (CED electronics, version 2). The accelerometry axis with the greatest overall amplitude was used for subsequent analysis. A high pass Butterworth filter (corner 2) was applied and then a Fourier transform of the signal was derived. The dominant frequency was determined by the peak of the frequency spectrum. Total power of the spectra between 1 and 30 Hz was used as a marker of tremor severity.

### Robotic Apparatus and Task

Participants were seated with their forehead supported on a headrest. Their semipronated right hand gripped a manipulandum underneath a horizontally suspended mirror. The mirror prevented direct vision of the hand and arm and showed a reflection of a computer monitor mounted above. The visual display comprised of a central 30 mm square which indicated the starting position, a circular cursor (5 mm diameter) representing the position of the manipulandum and a 10 mm square target at one of four radially arranged positions (45**°**, 135**°**, 225**°** or 315**°**), 80 mm from the starting position. The start of the trial was indicated by the appearance of the target. Subjects were instructed to ‘shoot’ through the target with a smooth arm movement as this type of movement is thought to rely on feed-forward control; in this type of movement, angular error at the start of movement is similar to the angular error at the end of movement suggesting that online feedback processes do not pay a major role in this task [13, 14]. The cursor was visible throughout the trial. If movement duration was greater than 300 ms, the target changed from white to blue at the end of the trial indicating that the movement was too slow. After completion of the outward movement, participants were asked to relax and allow the robotic arm to return the arm to the central starting position. Once the cursor was re-centred the next target would appear.

**Fig. 1:**
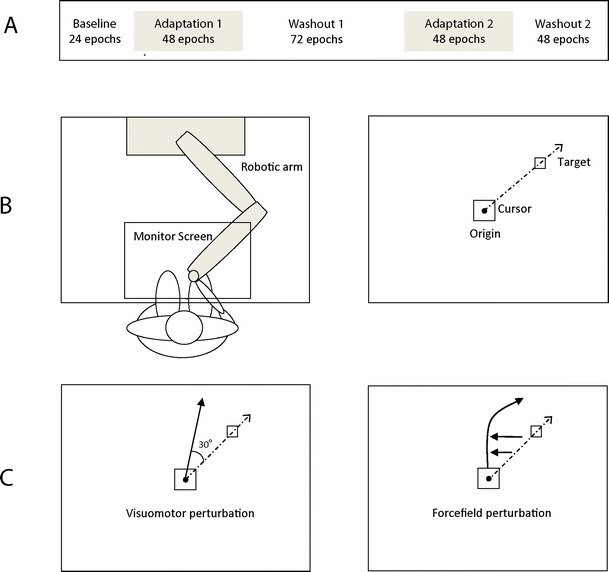
**a** Overview of experimental design. Each epoch consisted of four trials. **b** Robotic apparatus and baseline task. Subjects were seated and held a manipulandum with their right hand (see text for full description). Upon appearance of the target they were trained to make a shooting movement through this as accurately as possible (the perfect path is indicated on the diagram by the *dashed line*). **c** Schematic drawing of the perturbation conditions. The visuomotor condition consisted of a distortion of visual feedback by 30**°** in the *clockwise* (positive) or *anticlockwise* (negative) direction. The forcefield condition consisted of a *rightward* (positive) or *leftward* (negative) velocity dependent force applied to the robotic arm during movement (3 N/(m/s))

Participants familiarised themselves with the basic task by performing 25 trials during which verbal feedback was given to further explain the desired movement (data not analysed). Each participant then completed five experimental conditions in which baseline performance was assessed and then subjects were examined for their ability to adapt and washout both visuomotor and forcefield perturbations (Fig. 1). The visuomotor condition consisted of a distortion of visual feedback by 30**°** in the clockwise (positive) or anticlockwise (negative) direction. The forcefield condition consisted of a rightward (positive) or leftward (negative) velocity dependent force applied to the robotic arm during movement (3 N/(m/s)). The type of adaptation perturbation was counterbalanced such that if the first perturbation was positive visuomotor, the second perturbation was negative forcefield (giving four possible order combinations). The total time of the experiment was approximately 45 min.

### Kinematic Analysis

Hand position was sampled at a rate of 200 Hz. The outcome measures were angular error, movement duration and reaction time. Angular error was defined as the angular deviation from the ideal trajectory at the target perimeter. The start time (*t*_1_) of movement was defined as the time point at which 10 % of maximal velocity of that trial was reached. This avoided wrongly identifying small corrective movements of the cursor that were not the start of the shooting movement. The end of movement was defined as the time at which the target perimeter was first breached by subject movement (*t*_2_). Movement duration was the difference between these two values (*t*_2−_ *t*_1_). Reaction time was calculated as the difference between the time of target presentation (*t*_0_) and the start of movement (*t*_1_–*t*_0_). Trials that had an angular error >±45°, a movement duration <200 ms or >800 ms, or a reaction time <200 or >600, were excluded (in cervical dystonia 15.7 % of trials, in controls 14.3 %). Epochs of all kinematic variables were created by taking an average value across four consecutive trials.

The primary outcome, angular error, of the four conditions (visuomotor adaptation, visuomotor washout, forcefield adaptation, forcefield washout) was modelled using:

$$ Y=a+\kern0.5em  bex{p}^{\left(- cx\right)} $$

where *Y* represents the predicted angular error, *a* is an estimate of the plateau of the learning curve, *b* is an estimate of the maximal initial error (the *y*-intercept), *c* estimates the learning index for each condition and *x* is the epoch. The learning index is the percentage reduction in error for each epoch and thus can be used as a measure of the rate of adaptation and the rate of washout of perturbations. The adjusted *R* ^2^ value was calculated to analyse goodness of fit of the model. If *R* ^2^ was less than 0.4 (i.e., explained less than 40 % of variation) then the individual’s data for that perturbation were excluded from further group analysis (13 % excluded).

### Statistical Analysis

SPSS (IBM SPSS Statistics, v21), Excel (Microsoft Excel for Mac 2011, v14.3.7) and Matlab (R2011b) were used for data analysis, and all data are given as mean ± standard error of the mean (SEM). G*Power 3 [15] was used for the power calculation. Learning indices were compared using *t* tests with Bonferonni correction for the four conditions (level of significance after correction 0.05/4). Reaction time and movement duration were compared between cervical dystonia and controls during the fast learning for each condition using analysis outlined in previous studies [14]. For each subject, a mean value was calculated during the initial rapid rate of learning such that for the baseline block (total of 24 epochs), epochs 2–6 were averaged and for the adaptation and washout conditions (total of 48 epochs), epochs 2–11 were averaged [16]. Repeated measures analysis of variance (rmANOVA) were used to compare mean reaction time with the factors GROUP (control, dystonia) and CONDITION (baseline, visuomotor adaptation, visuomotor washout, forcefield adaptation, forcefield washout). This analysis was repeated for movement duration.

The severity of cervical dystonia as defined by the TWSTRS (both severity subscore and total) and learning index were correlated (Pearson’s correlation coefficient (*r*) and the *p* value are given). To examine for a potential relationship between tremor and adaptation, subjects were grouped into clinically apparent tremor and no tremor and *t* tests were performed to compare the learning index of the two groups for the adaptation and washout conditions. For patients with clinically apparent tremor, total power as an estimate of severity was correlated to the learning index for each adaptation/washout condition. Log transformation of total power was performed to normalise data which allowed the subsequent Pearson’s correlation.

## Results

### Summary

Rates of adaptation (learning) in cervical dystonia were identical to healthy controls in both visuomotor and forcefield tasks. Furthermore, the ability to adapt was not clearly related to clinical features of dystonic head tremor.

### Adaptation

All subjects completed the experiments. Mean age and variability were matched between groups (control mean 56.0 years (±2.46), patient mean 60.3 years (±2.80), *t*(36) = 1.15, *p* = 0.255). One patient and one control were excluded from all further analysis due to consistently low movement durations (necessary due to velocity dependent forcefield). In addition, tremor data were not available (NA) for two patients due to a technical failure.

**Fig. 2:**
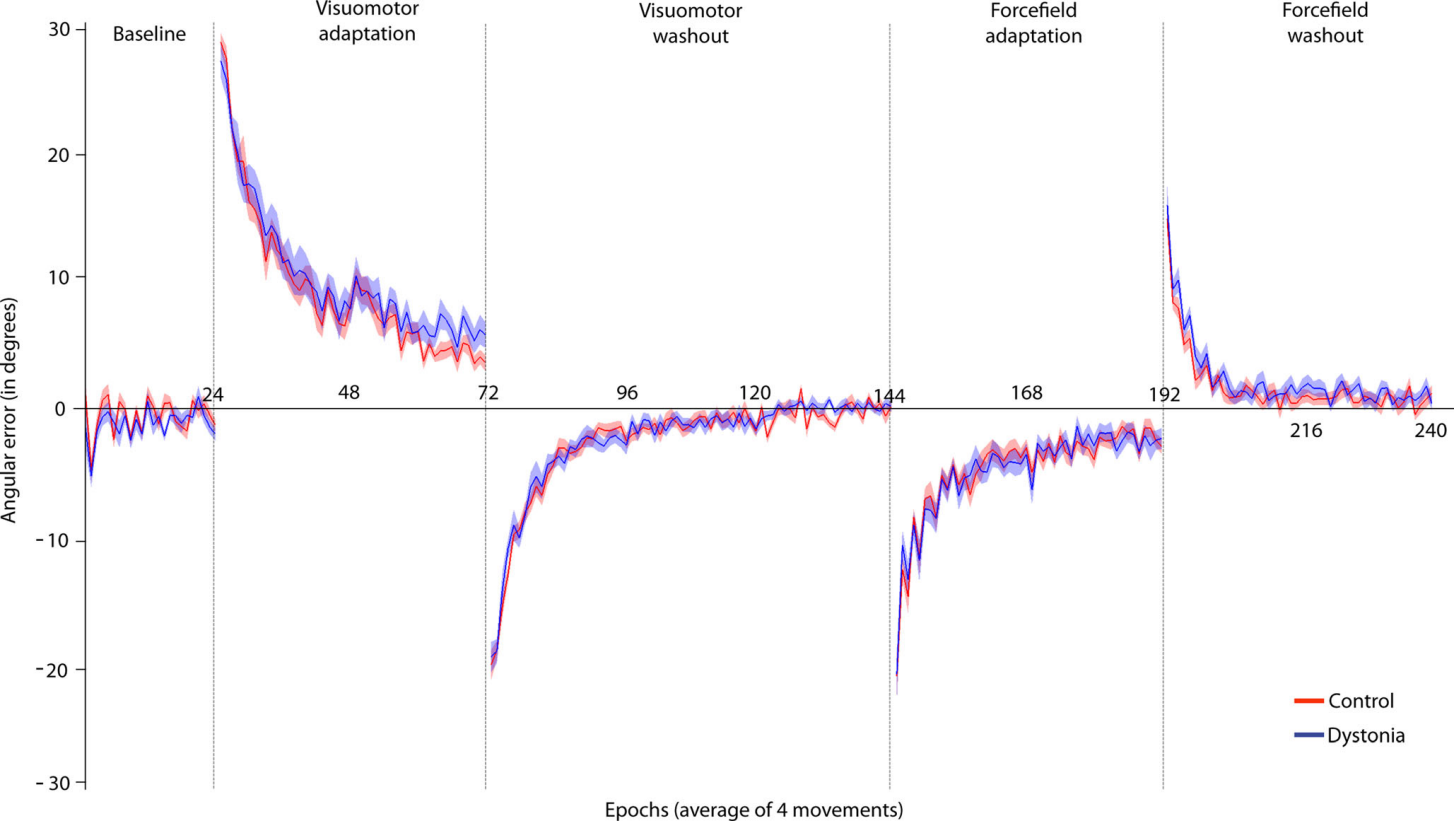
Group data for angular error across baseline, adaptation and washout conditions. All data sorted into the same order for figure (experimental design consisted of four possible order combinations.) Control data shown in *red*; cervical dystonia data shown in *blue*. The *solid line* indicates the mean and the *shaded regions* the standard error

**Fig. 3:**
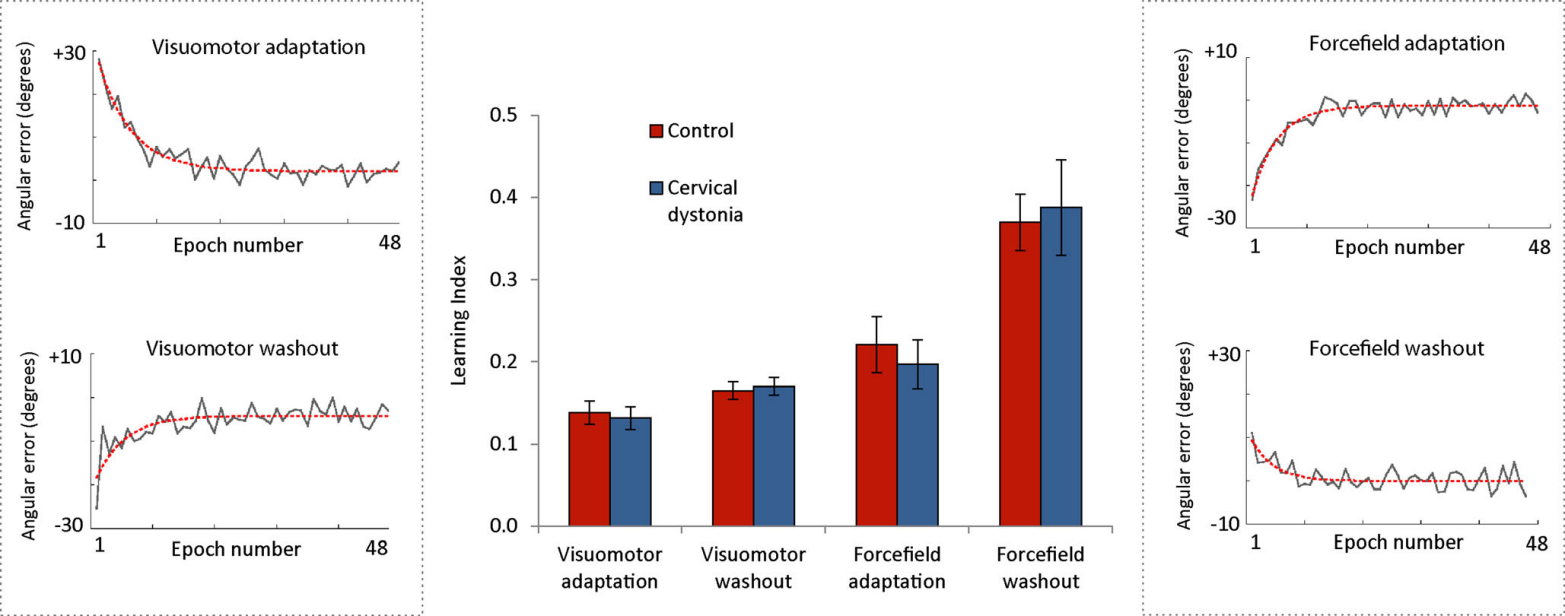
The performance of the model for an individual patient, and its ability to capture the rate of learning for each condition, is shown in the *boxes* either side of the bar chart. The *central bar chart* plots mean learning index for the two groups with the standard error of the mean indicated by the *error bars*. *Red (left bar)* control. *Blue (right bar)* cervical dystonia

In Fig. 2, the angular error for the five conditions is shown for controls (red) and subjects with dystonia (blue). Visually, rates of learning were very similar between groups. To compare rates of learning, an exponential model was applied to each participant’s data for the four adaptation conditions. In Fig. 3, the angular error of one subject (epochs of 4) is indicated by the solid grey line and the generated model by the dashed red line. It can be seen that the model accurately captures the slope of the curve in each condition which was the main parameter of interest (learning). To be included in analysis, the models adjusted *R* ^2^ had to exceed 0.4. The number of exclusions is indicated in Table 2. Crucially, out of 152 models, 87 % reached this criterion. The mean *R* ^2^ of the included models were not significantly different between groups: visuomotor adaptation *t*(22.7) = −0.770, *p* = 0.449, visuomotor washout *t*(33.7) = −0.525, *p* = 0.603, forcefield adaptation: *t*(32) = 0.413, *p* = 0.683, forcefield washout *t*(25) = 0.187, *p* = 0.853.

**Table 2:** Model parameters for adaptation and washout for both visuomotor and forcefield pertubations. The number of inclusions out of a total possible of 19 is detailed in the table (*n*) and the mean *R* ^2^ of the remaining group given

	Visuomotor adaptation					Visuomotor washout					Forcefield adaptation					Forcefield washout				
	Plateau (a)	Maximal error (b)	Learning index (c)	*n*	mean *R* ^2^	Plateau (a)	Maximal error (b)	Learning index (c)	*n*	mean *R* ^2^	Plateau (a)	Maximal error (b)	Learning index (c)	*n*	mean *R* ^2^	Plateau (a)	Maximal error (b)	Learning index (c)	*n*	mean *R* ^2^
Controls	4.02	28.0	0.139	18	0.80	1.49	23.8	0.164	18	0.81	3.02	22.7	0.222	17	0.61	0.849	20.2	0.369	15	0.57
CD	5.15	25.2	0.131	17	0.77	1.56	23.4	0.168	18	0.80	3.07	20.4	0.200	17	0.62	0.971	25.5	0.389	12	0.58

The primary outcome, rate of adaptation and/or washout (mean learning indices) were not statistically different in any of the four conditions: visuomotor adaptation *t*(33) = −0.396, *p* = 0.695, visuomotor washout *t*(34) = 0.287, *p* = 0.776, forcefield adaptation *t*(32) = −0.553, *p* = 0.584 and forcefield washout *t*(25) = 0.254, *p* = 0.801. The profile was also remarkably similar in that for both groups (central bar chart Fig. 3): visuomotor adaptation was slower than forcefield adaptation as evidenced by smaller learning indices, washout of the visuomotor perturbations had a rate comparable to visuomotor adaptation and rates of forcefield washout were greater than the rates of forcefield adaptation. The plateau and maximal error for each condition were also similar in dystonia to controls (values given in Table 2, no statistical difference found).

### Kinematic Variables

There were no significant differences between groups for reaction time (Table 3). RmANOVA revealed no significant difference for GROUP *F*(1,18) = 0.150, *p* = 0.703 or GROUPxCONDITION F(4,72) = 0.633, *p* = 0.604. There was a significant effect of CONDITION *F*(4,72) = 14.098, *p* < 0.001 however Tukey post-hoc tests following a one-way ANOVA were not significant.

**Table 3:** Mean reaction time and movement duration (with SEM) for patients and controls

	Reaction time (ms)		Movement duration (ms)	
	Control	Cervical dystonia	Control	Cervical dystonia
Baseline	459 (23)	405 (21)	305 (11)	315 (11)
Visuomotor adaptation	446 (22)	407 (16)	290 (7.7)	289 (9.2)
Visuomotor washout	414 (20)	413 (18)	288 (6.4)	291 (8.3)
Forcefield adaptation	420 (22)	424 (23)	274 (9.4)	286 (8.0)
Forcefield washout	387 (17)	439 (24)	278 (7.1)	289 (6.8)

There were no significant differences between groups for movement duration. RmANOVA did not show an effect of GROUP *F*(1,18) = 0.641, *p* = 0.434 or GROUP×CONDITION *F*(4,72) = 0.379, *p* = 0.823. A significant effect of CONDITION was observed *F*(4,72) = 6.879, *p* < 0.001. One-way ANOVA with Tukey post hoc analysis showed that this effect was due to a significant difference between baseline (highest movement duration) and forcefield adaptation (*p* = 0.006) and forcefield washout (*p* = 0.022).

### Clinical Correlations

Firstly, the severity of cervical dystonia was assessed for any relationship to rates of adaptation and washout. Both the severity subscore of the TWSTRS (visuomotor adaptation *r* = −0.22, *p* = 0.401; visuomotor washout *r* = −0.02, *p* = 0.938; forcefield adaptation *r* = −0.04, *p* = 0.887; forcefield washout *r* = −0.07, *p* = 0.832) and the total score of the TWSTRS (visuomotor adaptation *r* = 0.13, *p* = 0.612; visuomotor washout *r* = 0.7, *p* = 0.770; forcefield adaptation *r* = 0.002, *p* = 0.993; forcefield washout *r* = 0.14, *p* = 0.658) were correlated, and no relationships were found.

Eleven of the 19 patients had clinically apparent head tremor. The dominant frequency of the tremor had a mean of 5.02 Hz (SEM 0.331 Hz, range 3.1 to 7.5 Hz) in keeping with previous observations. As there was no clear grouping of tremor severity based objectively on total power of tremor alone, we divided subjects into those with clinically apparent head tremor (11 patients) and those without apparent head tremor (8 patients). The learning indices for these two groups were comparable in all four tasks with no significant difference seen (Table 4). In the patients with tremor, there was no correlation between tremor severity (log of total power) and the learning indices of each of the four conditions: visuomotor adaptation (*r* = −0.14, *p* = 0.764), visuomotor washout (*r* = 0.24, *p* = 0.562), forcefield adaptation (*r* = 0.25, *p* = 0.557), forcefield washout (*r* = −0.47, *p* = 0.347)*.*

**Table 4:** Comparison of the mean values of learning indices in patients with and without tremor. Learning indices were only included in comparison if the model suitably fitted the data (*R* ^2^ > 0.4). Eleven of the 19 patients had clinically apparent tremor

Condition	Group	*n*	Learning index mean (SEM)	*t* test
Visuomotor adaptation	No tremor	8	0.108 (0.019)	*t*(15) = 1.58, *p* = 0.134
	Tremor	9	0.152 (0.021)	
Visuomotor washout	No tremor	8	0.182 (0.018)	*t*(16) = −1.18, *p* = 0.280
	Tremor	10	0.158 (0.013)	
Forcefield adaptation	No tremor	7	0.189 (0.049)	*t*(15) = 0.188, *p* = 0.853
	Tremor	10	0.201 (0.042)	
Forcefield washout	No tremor	5	0.412 (0.048)	*t*(10) = −0.255, *p* = 0.804
	Tremor	7	0.373 (0.125)	

## Discussion

In this study, we have demonstrated that motor adaptation in cervical dystonia is identical to healthy controls in two tasks which test visual and proprioceptive sensorimotor integration. These data support preserved cerebellar function within this domain. We discuss these results in the context of recent dystonia research, which increasingly declares an important role for the cerebellum in the pathophysiology of dystonia.

### Evidence for Cerebellar Involvement in Dystonia

Work in animal models strongly supports a causal cerebellar contribution in the genesis of dystonia. For example, in murine animal models, a dystonic-like condition can be provoked (excitation) or eliminated (inhibition or cerebellectomy) by modulating the activity of the cerebellum [17] and genetically modified animal models are increasingly sophisticated in their ability to probe and implicate the cerebellum [18].

In humans, clinical data suggests that structural and degenerative disorders of the cerebellum can cause secondary forms of dystonia [19]. However, secondary dystonia, by definition, has differences to primary dystonia in which no gross structural pathology is observed. In addition, such disorders rarely selectively involve only the cerebellum, and pathology within the cerebellum may evoke compensatory change in a multitude of interconnected regions. Therefore, parallels drawn between this clinical data and primary dystonia should be tentative. In primary dystonia, subtle structural and functional abnormalities of the cerebellum and its communicating tracts have been consistently demonstrated with a range of imaging techniques (for review see [20–22]). Some electrophysiological data also point to cerebellar abnormalities in dystonia. Cerebellar brain inhibition (CBI) of the motor cortex was reduced in a small number of patients with focal hand dystonia [23]. In addition, the ability to acquire eye blink conditioning was reduced in cervical and focal hand dystonia, suggesting impaired cerebellar function [24]. Interestingly, this deficit in conditioning could be improved by further practice or inhibitory cerebellar stimulation, suggesting that cerebellar ‘dysfunction’ in dystonia is a dynamic process [25]. To date, deficits in CBI have not been replicated in a larger study nor investigated in other subgroups of dystonia and we have recently shown that eye blink conditioning is intact in the genetic dystonias DYT1 and DYT6 (submitted). Another line of investigation examining cerebellar function in dystonia has been the study of motor tasks which require intact cerebellar function. In DYT1 dystonia, both manifesting and non-manifesting subjects are impaired in sequence learning in which the sequential order targets is learnt [26, 27] and functional imaging demonstrated overactivity of the left cerebellar cortex (whilst subjects moved the right arm) [27, 28]. However, sequence learning recruits many brain regions including the basal ganglia, and the overactivation of the contralateral cerebellar hemisphere to hand movement (cerebellar control is ipsilateral) makes the functional significance of these findings in dystonia difficult to elucidate. Recently, it has been shown that sequence learning is normal in cervical dystonia [29].

### Motor Adaptation and Dystonia

Motor adaptation is a task commonly used, across species, to directly examine cerebellar function [14, 30]. An environmental perturbation introduces a movement error requiring subjects to adapt their performance of a task. The sensory prediction error (how the actual sensory movement outcome differed from the predicted sensory movement outcome) is used to update subsequent motor performance, with this type of learning being strongly dependent on the cerebellum [5]. Interestingly, the cerebellum has not only been linked to the formation of forward models which predict the sensory outcomes of motor commands; it may be that the cerebellum has a role in forming cognitive predictions for non-motor cerebellar functions such as language [31, 32]. This argument is supported by the highly conserved structure of the cerebellar microanatomical architecture, which is thought to imply that the computational qualities of cerebellar cortex remain constant [32, 33].

Patients with myoclonus dystonia (caused by mutations of the SGCE gene, DYT11) have been shown to have impaired saccadic adaptation [34]. It is difficult to dissociate in this condition whether the predominant phenotype of myoclonus dominates the neuroanatomical findings or whether the milder dystonia also has a role. In primary dystonia, motor learning/adaptation has been examined in focal hand dystonia using a joystick task [35]. Each trial had a different visuomotor perturbation, and a different position of the target and subjects were asked to correct their movement during each trial. No impairment in motor learning was demonstrated but there was impaired retention. However, contrary to the author’s conclusions, this suggests a change in the ability of the motor cortex to retain the new memory rather than a cerebellar deficit [14, 35]. Our data in cervical dystonia builds on previous work that we performed with a more simplistic visuomotor adaptation task [29]. This current study differs in that we used a purpose built robot which required larger more complex movements recruiting proximal arm and shoulder muscles. We also used a shooting paradigm which does not allow for online correction and modelled data in a manner which we believe optimally assesses for differences in adaptation. The forcefield condition is more relevant to dystonia in which subtle proprioceptive deficits have been described [36]. Furthermore, visuomotor and forcefield adaptation examine distinct (and common) regions of cerebellar function. Within the anterior lobe of the cerebellum, which contains one of the two body representations within the cerebellum, lobules IV and V are thought to be more important for the forcefield task and lobule VI is more important for visuomotor adaptation [8]. Regions in the posterolateral cerebellum (crus I and II) are thought to be required for both tasks [22]. This analysis of the two perturbations with a large number of patients leads us to confidently conclude that motor adaptation is normal in cervical dystonia.

### Significance of our Results

How do our results link in with the growing body of evidence which implicates the cerebellum in the pathophysiology of dystonia? Certainly for cervical dystonia, if there is cerebellar dysfunction, the nature and extent of cerebellar dysfunction remain to be established. Normal cerebellar adaptation in cervical dystonia is in contrast to deficits in eye blink conditioning within the same subtype of dystonia [24, 25]. Both paradigms are well-characterised paradigms in their assessment of cerebellar function. Perhaps the deficit in eye blink conditioning, with its greater reliance on millisecond timing, signifies that timing is the specific cerebellar deficit in dystonia? This viewpoint links well with abnormalities in temporal discrimination and other timing tasks that have been found within the millisecond range in focal dystonia [37–39]. One counter to this argument is the observation that all movement parameters were normal in the current study, and each parameter has a millisecond timing requirement. Furthermore, a deficit in millisecond timing capabilities could potentially impair the generation of sensory prediction errors, which would include time as one of their dimensions.

The normal performance in these adaptation tasks that required use of both visual and proprioceptive input was of interest. Although visual processing is normal in cervical dystonia, previous studies have described deficits in proprioceptive tasks dystonic subjects are less sensitive at detecting passive movements of the fingers [40] and arms are abnormal in their perception of the vibration induced illusion of movement (which is induced by stimulating muscle spindles with a vibration stimulus) [36, 41, 42]. How can performance in our tasks be normal in the face of such obvious deficits? One possibility is that tests of proprioceptive sensation are mostly static tasks whereas ours were dynamic, involving sensation during active movement. Furthermore, the psychophysical tasks described above require sensory processing and decision making at many levels of the nervous system and some of these are likely to be distinct to networks involved in implicit motor tasks. For example, higher order/consciously regulated elements of decision making could have a greater influence on psychophysical tasks.

The question of whether movement in the asymptomatic arm of patients with cervical dystonia is entirely normal perhaps remains to be definitively answered with future experimental work. Some have described abnormalities in kinematic variables recorded during reaching studies similar to the task used in this article (movement time was not matched between groups and thus some of this data is difficult to interpret [40]) and electrophysiologically, abnormalities in inhibition have been demonstrated at many levels of the nervous system concerned with the control of the arm musculature (e.g., abnormal reciprocal inhibition of forearm muscles in cervical dystonia [43]). However, other studies in including ours suggest near normal motor performance [29]. Conservation of motor skill in the arms is the norm with most patients with cervical dystonia, and we argue that this is perhaps against a global movement deficit in the focal dystonias.

Our conclusions for dystonic tremor are more tentative. We did not find evidence to support a relationship between the ability to adapt and the severity of dystonic tremor. Secondly, splitting subjects into whether or not they had tremor did not reveal a group difference in rates of adaptation. The pathophysiology of dystonic tremor is poorly understood but many primary tremor models are thought to involve the cerebellothalamocortical network. Certainly, in patients with essential tremor, there seems to be multimodal evidence for pathological involvement of the cerebellum (structural imaging [44], functional imaging [45], eye movement analysis [46], deficits in eye blink conditioning [47] and motor adaptation [12].) Here, we have performed one of the first studies to examine the role of the cerebellum in the generation of dystonic head tremor and have not yet found a clear interaction. Our findings support studies that suggest different mechanisms between essential and dystonic tremor. For example, in essential tremor, the second agonist burst during ballistic movements is delayed and this finding is often ascribed to a lack of cerebellar prediction [48]. This delay in timing is not observed in patients with dystonic tremor [49].

A final implication of our results is that the preservation of adaptation, a type of motor learning, may have potential therapeutic implications. Adaptation could be used to reduce errors in dystonic movements, and this could translate into advances in physical therapy for dystonia [50].

A limitation of our study is the possibility that our task was insensitive to a deficit in adaptation. Perhaps errors were too large in our task to detect cerebellar dysfunction within a biologically relevant range. Against this is the observation that patients with cerebellar damage had an equal difficulty with small and large perturbation errors [51]. Furthermore, based on our mean and variance from the visuomotor adaptation condition (effect size 0.097) and assuming a power level of 0.8, we would need approximately 2,700 subjects in total in order to achieve a significant result. Therefore, we do not believe our null results are due to a lack of power. Another perhaps unavoidable limitation is that patients were receiving botulinum toxin injections (the mainstay of treatment for cervical dystonia). We tested patients when maximally symptomatic prior to injections but the long-term influence of botulinum injections on results cannot not be fully assessed in this or other studies that have used an identical approach.

## Conclusions

We have shown that adaptation, a fundamental computation of the cerebellum, is normal in cervical dystonia. Furthermore, the ability to adapt is not clearly related to clinical features of dystonic head tremor. These results have important implications in the current thinking of the pathophysiology of dystonia. It is important to progress from very general statements that implicate the cerebellum in the genesis of dystonia to a more specific evidence-based model. Future research should aim to critically and directly examine the extent and nature of cerebellar involvement in a hypothesis driven framework. The role of the cerebellum in this enigmatic disease perhaps remains to be proven.
